# Genomic insights into *Streptomyces albidoflavus* SM254: tracing the putative signs of anti-*Pseudogymnoascus destructans* properties

**DOI:** 10.1007/s42770-025-01740-8

**Published:** 2025-07-22

**Authors:** Ilia V. Popov, Michael L. Chikindas, Igor V. Popov

**Affiliations:** 1 Faculty “Bioengineering and Veterinary Medicine”, Don State Technical University, Rostov-on-Don, 344000 Russian Federation; 2 Health Promoting Naturals Laboratory, School of Environmental and Biological Sciences, Rutgers State University, New Brunswick, NJ 08901 USA; 3 Department of General Hygiene, I.M. Sechenov First Moscow State Medical University, Moscow, 119435 Russian Federation; 4 Division of Immunobiology and Biomedicine, Center of Genetics and Life Sciences, Sirius University of Science and Technology, Krasnodar Krai, 354340 Russian Federation

**Keywords:** *Streptomyces albidoflavus* SM254, *Pseudogymnoascus destructans*, White-nose syndrome, Comparative genomics

## Abstract

White-nose syndrome, caused by the psychrophilic fungus *Pseudogymnoascus destructans*, has devastated bat populations across North America. *Streptomyces albidoflavus* SM254 was previously reported to exhibit antifungal activity against this pathogen, but no comprehensive genomic characterization has been performed to date. Here, we analyzed 34 *S. albidoflavus* genomes, including the antifungal strain SM254 and 33 publicly available references, to investigate its metabolic potential and functional distinctiveness. Using pangenome reconstruction, phylogenomics, average nucleotide identity, and KEGG pathway profiling, we found that *S. albidoflavus* SM254 shares high nucleotide identity (> 99%) with five closely related strains but displays a unique combination of complete ethanol fermentation capacity and asparagine biosynthesis deficiency. These traits were exclusive to SM254 and may reflect adaptation to the oxygen-limited, nutrient-variable sediment environment. Functional annotation further revealed high completeness in central energy, redox, and stress-response pathways. Although direct antifungal mechanisms remain to be experimentally validated, *S. albidoflavus* SM254’s unique metabolic profile and ecological specialization suggest potential relevance in biocontrol contexts.

## Introduction

White-nose syndrome (WNS) is a devastating fungal disease affecting hibernating bat populations in North America [1]. Caused by the psychrophilic fungus *Pseudogymnoascus destructans* [2], WNS has led to the deaths of millions of bats since its discovery in 2006 [3], with mortality rates exceeding 90% in some colonies [4]. The disease disrupts hibernation, causing bats to deplete their fat reserves prematurely, leading to starvation and death [5]. This mass mortality has significant ecological implications, as bats play crucial roles in insect control and pollination, contributing substantially to ecosystem health and agriculture [6].

In 2016, Badalamenti et al. reported the complete genome sequence of *Streptomyces albidoflavus* SM254, a potent antagonist of *P. destructans*, isolated from copper-rich subsurface sediment collected at a depth of ~ 220 m in the Soudan Iron Mine (Minnesota, USA) [7]. This finding suggested the potential of SM254 in biocontrol strategies against WNS. However, since this initial report, there has been a lack of detailed studies exploring the genomic and functional attributes of SM254 that confer its antifungal properties.

More recently, comparative genomic analyses of *Streptomyces albidoflavus* strains isolated from rhizospheric soils have been conducted [8], focusing primarily on biosynthetic gene clusters responsible for antifungal secondary metabolites. Although *S. albidoflavus* SM254 was briefly introduced in that study, it was not a primary subject of the analysis, and its genomic features were not examined in detail. As a result, its potential antifungal mechanisms remain largely unexplored.

In this study, we present a comprehensive genomic analysis of *S. albidoflavus* SM254 to elucidate the genetic basis of its antagonistic activity against *Pseudogymnoascus destructans*. By examining its genome structure, functional annotations, and metabolic pathways, we aim to uncover the molecular determinants that contribute to its antifungal properties, thereby providing insights into potential biocontrol applications against WNS.

## Materials and methods

### Genome data collection

All available complete genomes of *Streptomyces albidoflavus* were retrieved from the NCBI RefSeq [9] database on 29/04/2025. The following query was used to identify target genomes:

*(“Streptomyces albidoflavus“[Organism] OR “Streptomyces albidoflavus“[All Fields]) AND “complete“[All Fields] AND (bacteria[filter] AND biomol_genomic[PROP] AND ref-seq[filter] AND is_nuccore[filter] AND (“5000000“[SLEN]: “20000000“[SLEN]))*

Genomes with annotated coding sequences were retained, resulting in a dataset of 34 *S. albidoflavus* reference genomes, including the antifungal strain *Streptomyces albidoflavus* SM254 (GenBank accession: NZ_CP014485).

### Pangenome construction and analysis

Pangenome analysis was conducted using the PanACoTA v.1.4.0 pipeline [10], which integrates genome quality control, pangenome computation, core genome alignment, and phylogenomic inference. The pangenome was computed using MMSeqs2 v.17 [11] clustering with a minimum amino acid sequence identity threshold of 90%. The resulting pangenome contained 11,638 single-copy gene families, of which 3,867 were present in all 34 genomes, constituting the strict core genome. Pangenome composition was assessed at the species-wide level and for the SM254 strain specifically. For global pangenome visualization, a composition-based donut chart was generated, representing core, accessory, and strain-specific gene clusters. For SM254, a strain-level bar chart illustrated the proportion of gene categories. All figures were generated in R v.4.4.0 using the following packages: ggplot2 [12], dplyr, tidyr, ggnewscale, ggrepel, and patchwork.

### Core genome phylogeny

The 3,867 core gene families were extracted and individually aligned using MAFFT v.7.526 [13] with default parameters. The resulting concatenated core genome alignment was subjected to model selection using ModelFinder within the IQ-TREE 2 suite [14]. The best-fit nucleotide substitution model based on the Bayesian Information Criterion was TVM + F + R5. A maximum-likelihood phylogenetic tree was inferred using IQ-TREE 2 v.2.4.0 [15] with 10,000 ultrafast bootstrap [16] replicates to assess branch support. Tree visualization and annotation were performed in R v.4.4.0 using the ggtree [17], ggtext, treeio, phangorn, ggnewscale, phytools, and viridis packages. Metadata associated with each genome (e.g., isolation year and country) were retrieved from NCBI RefSeq using the Phyloki v.0.5.51 (https://github.com/iliapopov17/phyloki) and used for tree annotation.

### Average nucleotide identity analysis

Pairwise genomic similarity among the 34 *Streptomyces albidoflavus* strains was calculated using FastANI v.1.34 [18]. The resulting Average Nucleotide Identity (ANI) matrix was visualized as a heatmap using seaborn [19] and matplotlib [20] in Python v.3.12. To enable focused comparative analysis within the phylogenetic clade containing *S. albidoflavus* SM254, a subset of six closely related genomes was selected: *S. albidoflavus* NBC_01621 (NZ_CP109294), NBC_01747 (NZ_CP109142), NBC_01673 (NZ_CP109224), SM254 (NZ_CP014485), NBC_01665 (NZ_CP109235), and NBC_01110 (NZ_CP108647). A separate ANI heatmap was generated for this six-strain subset to highlight fine-scale genomic variation within the clade.

### Functional annotation and metabolic profiling

Comprehensive functional annotation was performed on the six selected genomes using eggNOG-mapper v.2.1.12 [21] under default parameters. To streamline the workflow, the annotation process was encapsulated within a Snakemake pipeline [22]. Functional profiles based on KEGG Orthology annotations were analyzed using KEGGaNOG v.1.0 (https://github.com/iliapopov17/KEGGaNOG), a Python-based utility de-signed for processing eggNOG-mapper annotations. KEGG pathway modules associated with microbial metabolism and ecological functions were extracted and compared across the genomes. To visualize the results, multiple comparative plots were generated. The functional profile of *S. albidoflavus* SM254 was summarized in a bar chart. A heatmap was created to depict the completeness of KEGG pathways across the six strains. Additionally, a radar plot was used to compare the relative completeness of four representative pathways showing variability among strains. Finally, a correlation network based on functional similarity was constructed to assess the overall divergence in metabolic potential within the clade.

## Results

### Phylogeny and pangenome architecture of *Streptomyces albidoflavus*

**Fig. 1:**
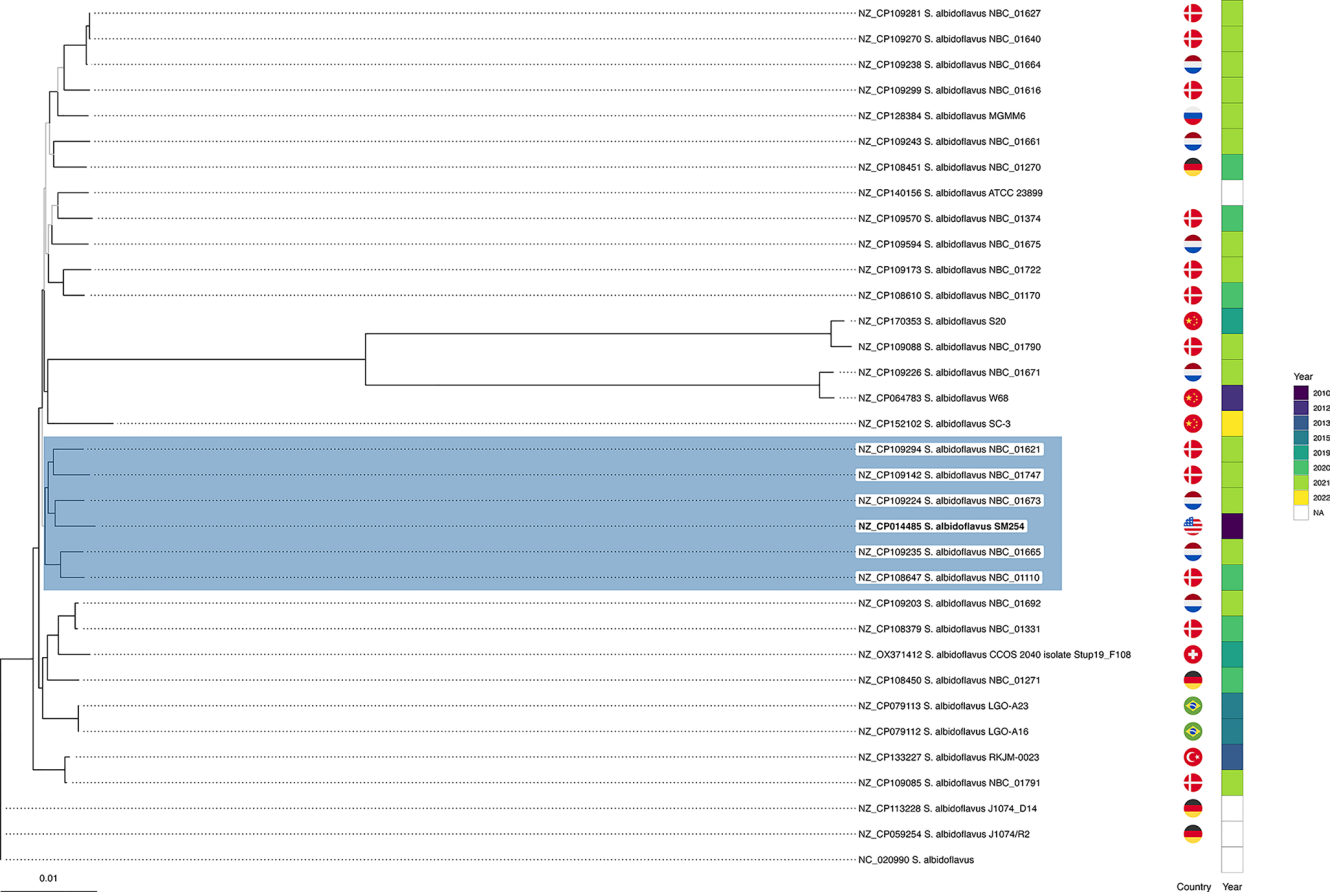
Phylogenomic structure of 34 *Streptomyces albidoflavus* genomes. Maximum-likelihood tree constructed from 3,867 concatenated core gene alignments under the TVM + F + R5 substitution model. The clade containing the SM254 strain is highlighted in blue, with SM254’s branch tip in bold. Branches with bootstrap support < 70 are shown in gray; those ≥ 70 are black. The country of isolation is indicated by national flags, and the year of isolation is represented as a color gradient. Scale bar denotes substitutions per site

The maximum-likelihood phylogenomic tree inferred from 3,867 concatenated core gene alignments revealed a coherent clustering pattern across the 34 strains (Fig. 1). A distinct clade comprising *S. albidoflavus* SM254 and five other strains– NBC_01621, NBC_01747, NBC_01673, NBC_01665, and NBC_01110– showed tight phylogenetic proximity. These strains were isolated across geographically and temporally distinct settings, including Denmark (2020–2021), the Netherlands (2021), and the United States (2010), indicating a globally distributed lineage with high genomic similarity. In contrast, four strains– S20, NBC_01790, NBC_01671, and W68– formed a divergent group, separated by a comparatively long phylogenetic branch length from the remaining strains. This divergence suggests deep lineage-level separation within the species.

**Fig. 2:**
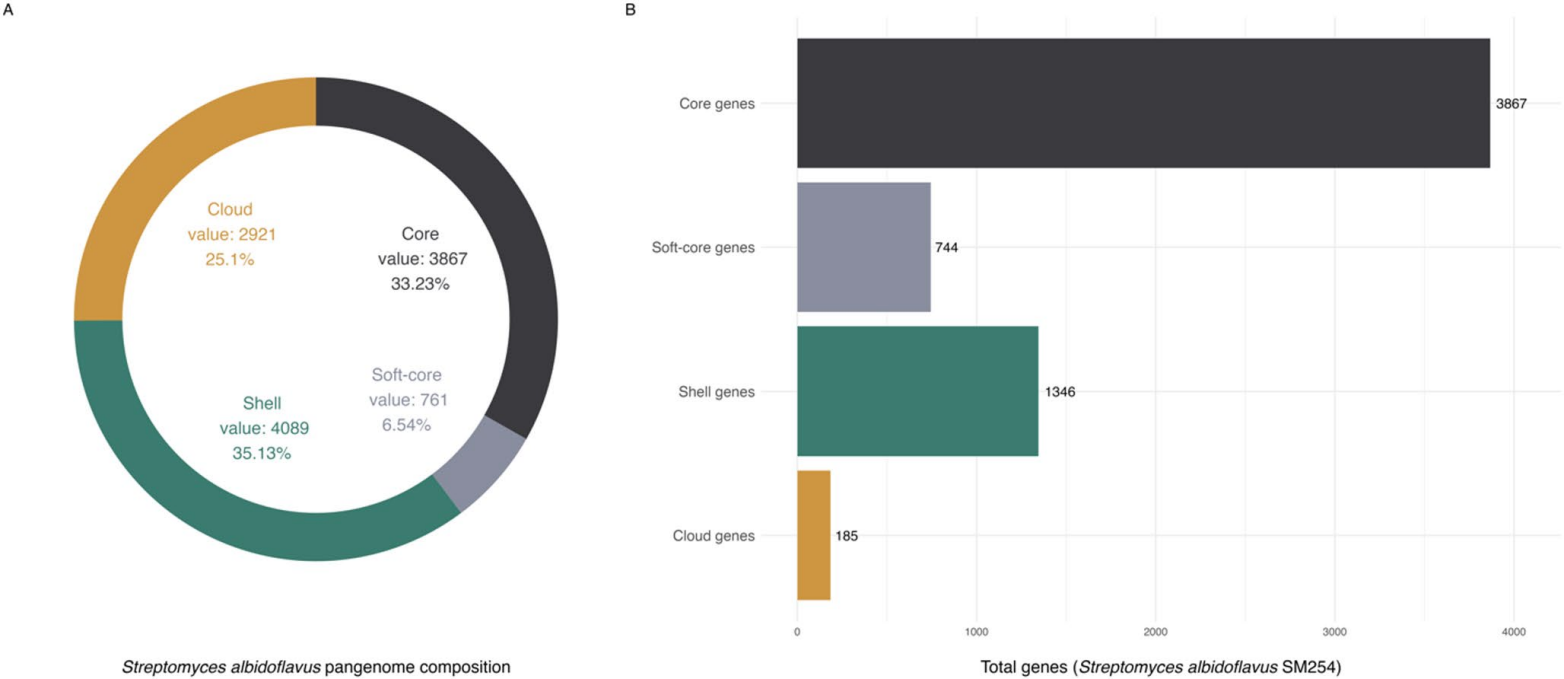
Pangenome composition of *Streptomyces albidoflavus*. (**A**) Species-wide pangenome structure of 34 *S. albidoflavus* genomes, showing the distribution of gene families into core (3,867), soft-core (761), shell (4,089), and cloud (2,921) categories. (**B**) Gene family distribution in the SM254 strain genome, consisting of 3,867 core, 744 soft-core, 1,346 shell, and 185 cloud genes

The species-wide pangenome of 34 *S. albidoflavus* strains comprised 11,638 single-copy gene families, of which 3,867 constituted the core genome shared by all strains. The soft-core genome (genes present in ≥ 32 strains) included 761 gene families, while the shell and cloud compartments consisted of 4,089 and 2,921 gene families, respectively (Fig. 2A). Within *S. albidoflavus* SM254, the genome contained the entire core set of 3,867 gene families, along with 744 soft-core, 1,346 shell, and 185 cloud gene families (Fig. 2B). These figures reflect both the conserved and accessory genomic repertoire of SM254 within the broader *S. albidoflavus* population.

### Average nucleotide identity-based genomic similarity

**Fig. 3:**
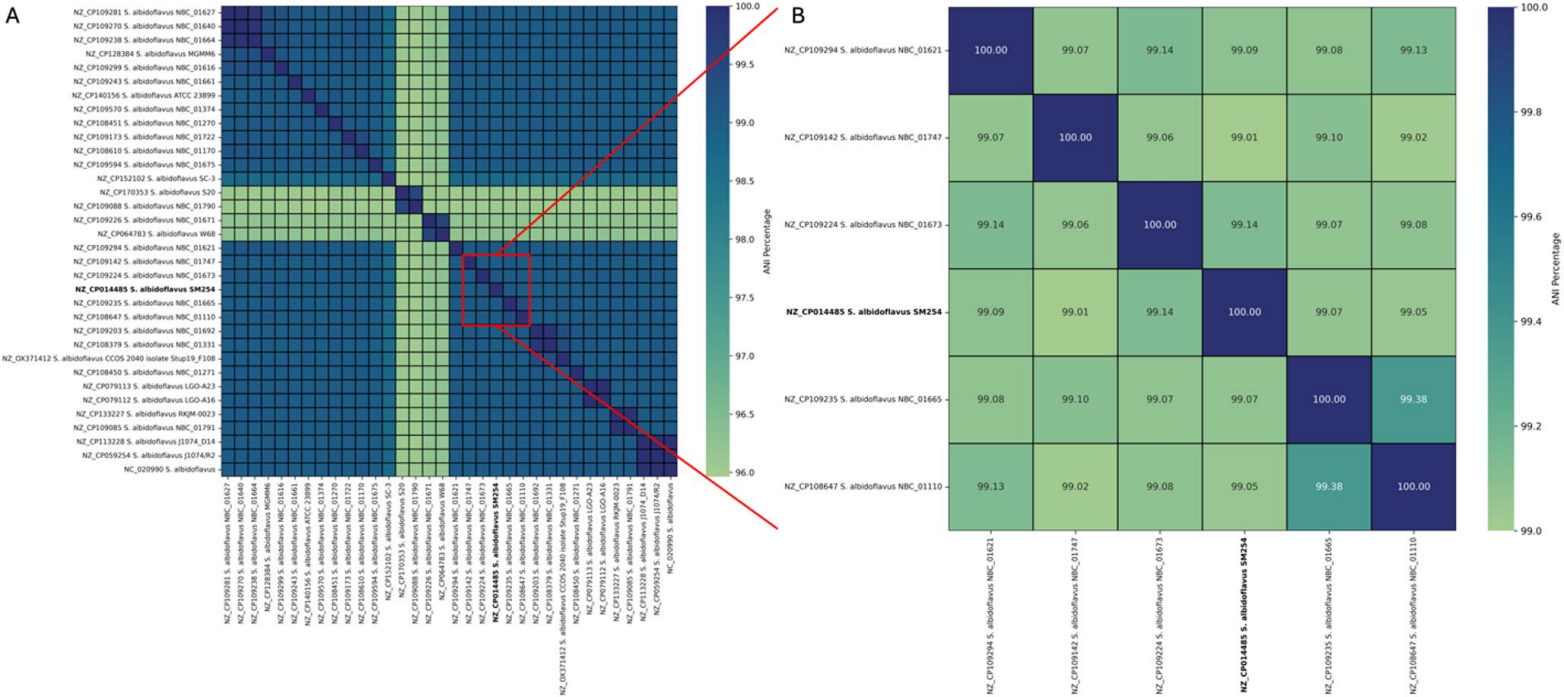
Pairwise average nucleotide identity (ANI) across *Streptomyces albidoflavus* genomes. (**A**) ANI heatmap showing genome-wide similarity across all 34 strains. (**B**) Subset heatmap highlighting ANI values among the six closest relatives of the SM254 strain. The SM254 genome is bolded for clarity. Color gradients represent ANI percentages

The pairwise ANI matrix (Fig. 3A) supported the phylogenomic structure. The same four divergent strains– S20, NBC_01790, NBC_01671, and W68– showed ANI values around 96% in comparison with other members of the species, consistent with their distinct phylogenetic position. All remaining strains exhibited high genomic identity, with ANI values near or exceeding 99%, suggesting a high level of genomic conservation among the main *S. albidoflavus* clade. Focusing on the SM254 clade, ANI values among the six members did not exceed 99.14%, with the exception of NBC_01665 and NBC_01110, which shared an ANI of 99.38% (Fig. 3B). This pattern confirms fine-scale genomic cohesion within the clade and provides a quantitative basis for delineating closely related but non-identical strains.

### Functional profile of *Streptomyces albidoflavus* SM254

**Fig. 4:**
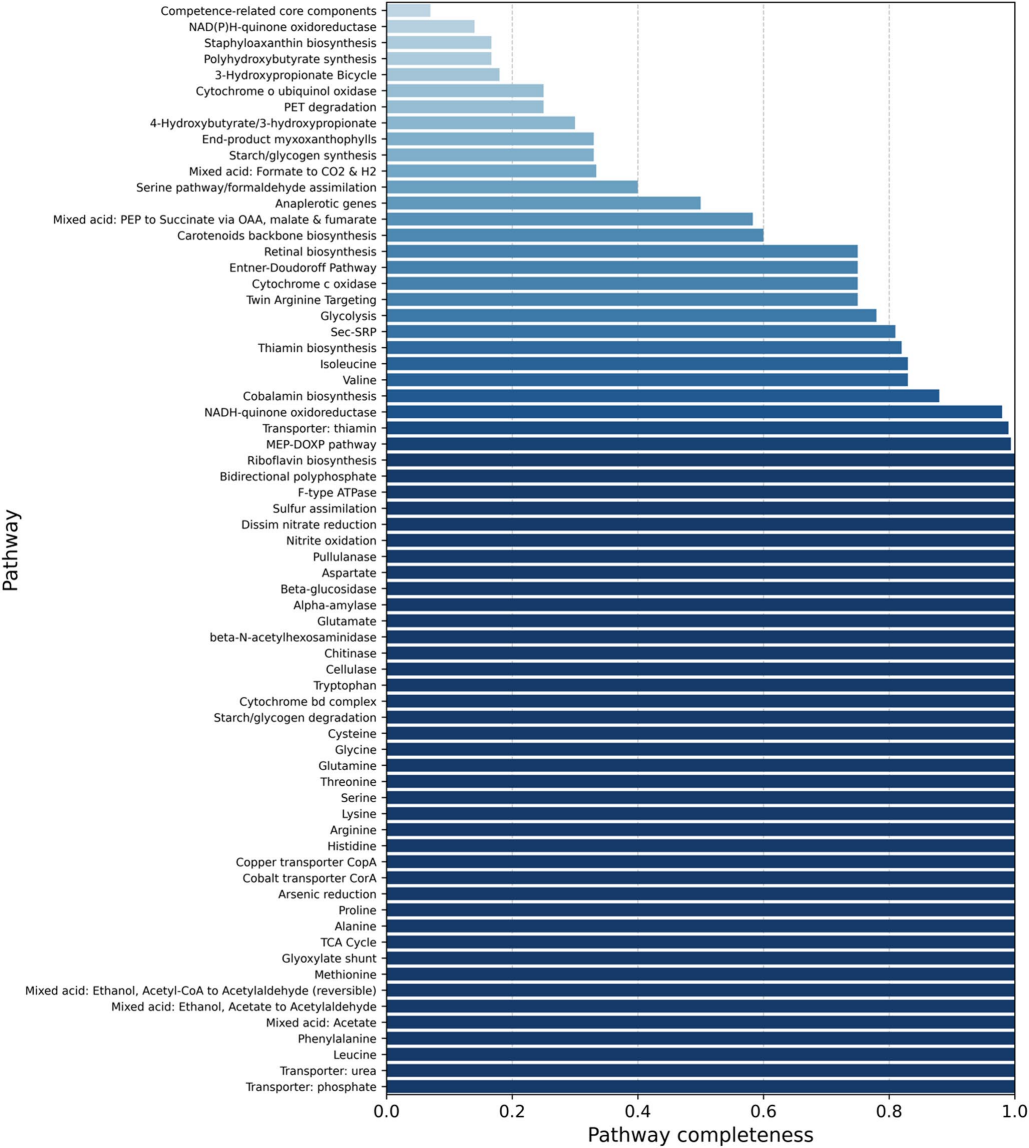
Functional pathway completeness in *Streptomyces albidoflavus* SM254. Barplot showing the KEGG-based completeness of 74 annotated metabolic pathways with non-zero completeness values, ranging from partial to fully complete (0 < completeness ≤ 1)

The metabolic reconstruction of *S. albidoflavus* SM254 revealed high completeness across core energy metabolism and biosynthetic pathways, as summarized in Fig. 4. The glycolysis module was 78% complete, whereas the gluconeogenesis pathway was not detected. The tricarboxylic acid cycle was fully complete (1.0), and NADH-quinone oxidoreductase reached 98% completeness. In contrast, alternative NAD(P)H oxidoreductase variants and sodium-coupled NADH oxidoreductases were either partially complete (14%) or absent.

The aerobic respiratory chain was well represented. SM254 encoded a complete F-type ATP synthase complex (1.0) and had a partially complete cytochrome c oxidase module (75%). In contrast, the V-type ATPase and Na+-translocating NADH: ubiquinone oxidoreductase modules were absent.

Central carbon metabolism pathways were well developed. The pentose phosphate pathway reached 92% completeness, and the Entner-Doudoroff pathway was fully complete (1.0). Additionally, acetate and acetyl-CoA conversion, citrate fermentation, and glyoxylate shunt modules were detected with moderate to high completeness. The ethanol fermentation pathway converting acetyl-CoA to acetaldehyde (mixed acid type) was fully complete (1.0), indicating the genetic potential for reversible ethanol metabolism under variable redox conditions.

Several biosynthetic pathways associated with amino acids, cofactors, and cell wall components were also well represented. The peptidoglycan biosynthesis module was complete, and pathways involved in fatty acid synthesis and NAD(P) cofactor metabolism showed completeness values near or above 90%. Multiple modules relevant to terpenoid and isoprenoid precursor synthesis were detected: the MEP-DOXP pathway was nearly complete (99.4%), while the mevalonate pathway was absent.

Polyhydroxybutyrate synthesis and staphyloxanthin biosynthesis were both detected but only partially complete (16.7% each). Among carotenoid backbone and pigment-related modules, moderate completeness values were observed for carotenoid backbone biosynthesis (60%) and myxoxanthophyll synthesis (33%), while other pigment pathways (e.g., astaxanthin, nostoxanthin) were absent.

Polyphosphate metabolism was prominently represented: the bidirectional polyphosphate pathway was fully complete (1.0), supporting metabolic flexibility under phosphate stress.

Overall, *S. albidoflavus* SM254 exhibited a metabolically versatile profile with complete or near-complete central energy pathways, respiratory components, and biosynthetic modules linked to cofactors and stress resilience. The partial presence of pigment and polymer synthesis modules suggests strain-specific variation in secondary metabolism.

### Strain-level variation in functional pathways

**Fig. 5:**
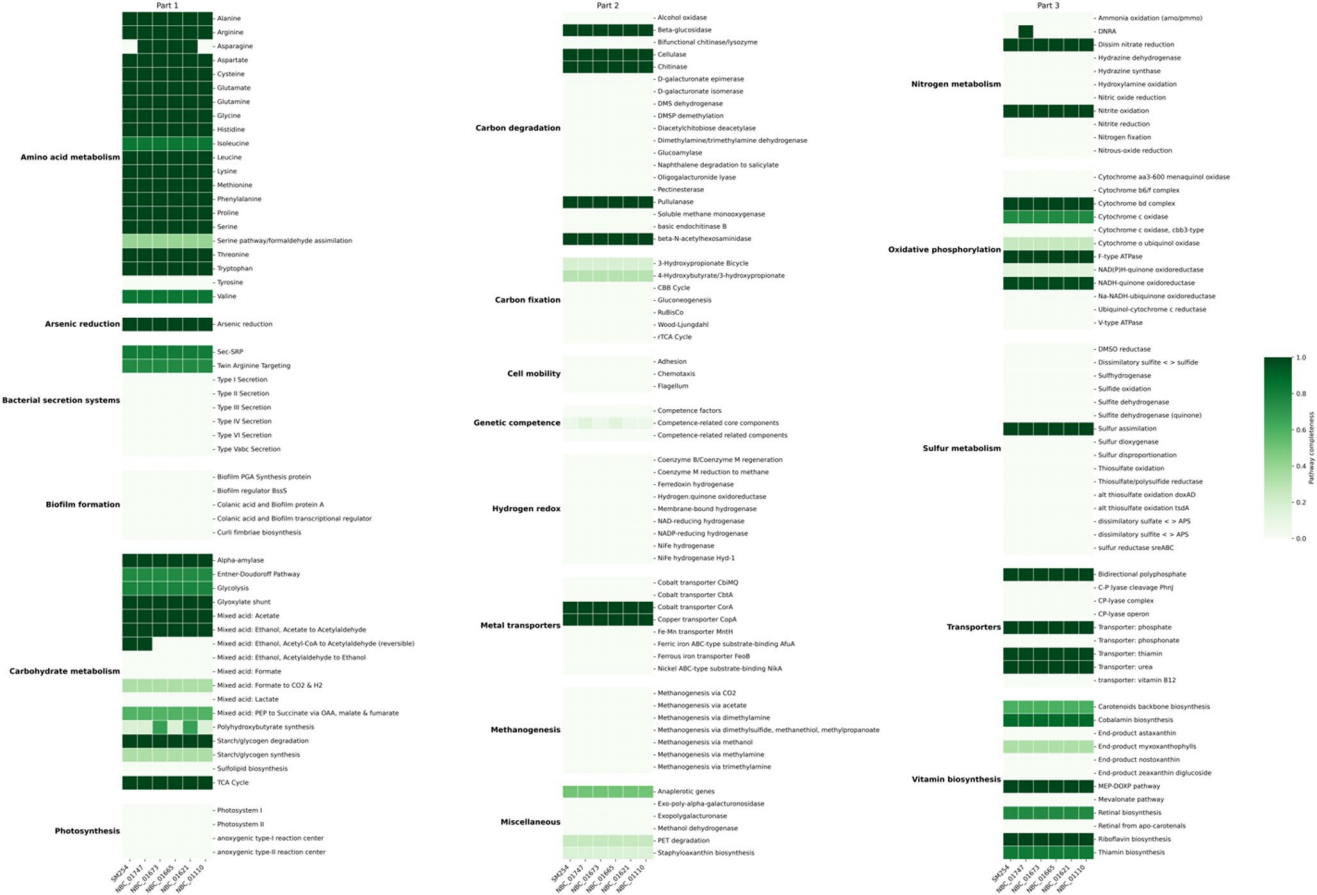
Strain-level variation in functional pathway completeness among *Streptomyces albidoflavus* strains. Heatmap displaying the completeness of annotated metabolic and ecological pathway modules across SM254 and its five closest relatives. Pathways are grouped by functional category (e.g., amino acid metabolism, oxidative phosphorylation, metal transporters) for clarity. Despite high ANI (> 99%), several differences in pathway presence and completeness were observed, highlighting fine-scale metabolic divergence

**Fig. 6:**
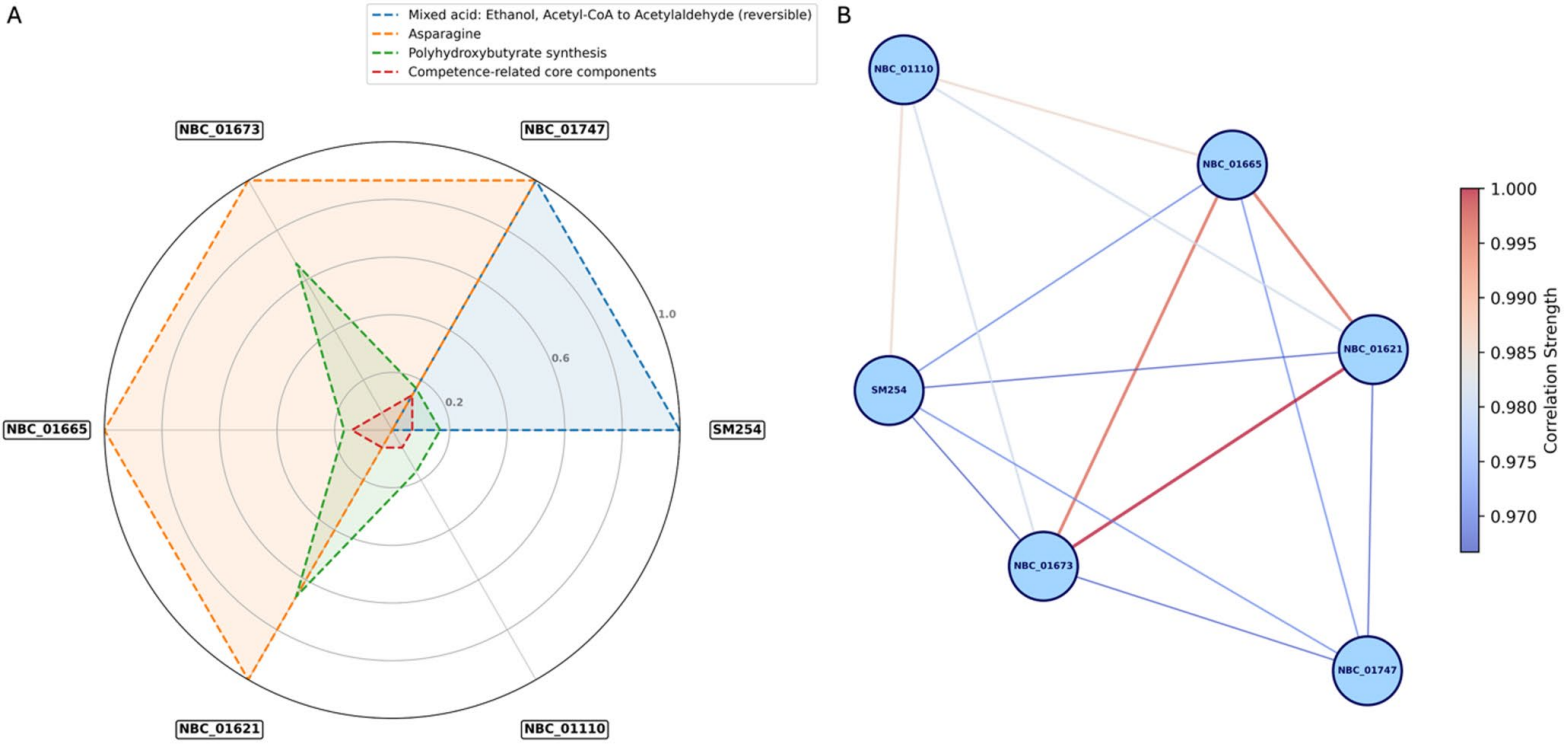
Functional divergence within the SM254 clade of *Streptomyces albidoflavus*. (**A**) Radar plot com-paring completeness values of four KEGG pathways across SM254 and its five closest relatives: ethanol fermentation (Mixed acid: Ethanol, Acetyl-CoA to Acetylaldehyde (reversible), asparagine biosynthesis, polyhydroxybutyrate synthesis, and competence-related core components. Each axis represents pathway completeness from 0 to 1. (**B**) Correlation network based on pairwise similarity of functional pathway profiles among the same six strains. Edge width and color in-tensity reflect correlation strength, with bolder lines indicating higher similarity. The network highlights fine-scale functional divergence within a group of genomes sharing > 99% ANI

Despite the high nucleotide identity observed among *S. albidoflavus* SM254 and its five closest relatives (ANI > 99%), several notable differences were detected in their functional repertoire, as summarized in Fig. 5. Four KEGG pathways– ethanol metabolism, asparagine biosynthesis, polyhydroxybutyrate synthesis, and competence-related core components– exhibited variable completeness among strains (Fig. 6A), highlighting minor functional variation within a highly conserved phylogenetic clade.

The pathway “Mixed acid: Ethanol, Acetyl-CoA to Acetylaldehyde (reversible)” was exclusively complete in SM254 and NBC_01747 (completeness = 1.0), but entirely absent in the other four strains. This distribution suggests potential differences in redox balance or alternative fermentative capacity, despite the near-identical genome content across the group.

Asparagine biosynthesis was fully complete (1.0) in NBC_01665, NBC_01621, NBC_01673, and NBC_01747, but entirely absent in SM254 and NBC_01110. This variation in amino acid metabolism may reflect regulatory or environmental adaptation, as all genomes encode other core amino acid biosynthetic modules with high completeness.

In the polyhydroxybutyrate synthesis module, NBC_01621 and NBC_01673 had higher predicted completeness (0.667) compared to SM254, NBC_01665, NBC_01747, and NBC_01110, which all showed lower values (0.167). These differences may indicate variations in carbon storage strategies or polymer synthesis capacity among strains.

Finally, the module associated with competence-related core components was consistently incomplete but varied in predicted gene content. NBC_01665 and NBC_01747 each reached 0.14 completeness, while all other strains, including SM254, had lower values ranging from 0.07 to 0.14. Although the module was incomplete in all cases, the observed variation suggests differential retention or decay of competence machinery within the clade.

A fifth pathway, DNRA: Nitrate to ammonia (dissimilatory), was detected only in a single strain and was therefore excluded from the radar plot presented in Fig. 6A.

These functional discrepancies are particularly noteworthy given the overall genomic similarity of the strains analyzed. The presence or absence of specific metabolic capabilities—such as ethanol fermentation or amino acid biosynthesis– within a clade of strains with > 99% ANI underscores the importance of fine-scale functional annotation in revealing biologically meaningful diversity.

A correlation network based on pairwise metabolic profile similarity further illustrated these fine-scale functional differences among strains (Fig. 6B). The network was constructed using pathway completeness-based correlation coefficients derived from the KEGG module annotations. Most strains in the SM254-associated clade showed strong mutual similarity, with pairwise correlation values consistently exceeding 0.96. The tightest relationships were observed between NBC_01621 and NBC_01673 (correlation = 1.0), as well as between these two strains and NBC_01665 (0.996).

Despite the overall coherence of the network, *S. albidoflavus* SM254 displayed reduced similarity relative to the rest of the clade. Its correlation with NBC_01621 and NBC_01673 was 0.966, while the similarity with NBC_01665 and NBC_01747 was marginally higher at 0.970 and 0.971, respectively. The highest correlation involving SM254 was with NBC_01110 (0.985), suggesting a closer functional resemblance between these two strains.

NBC_01747 and NBC_01110 formed weaker connections to each other (0.957) and to the rest of the network, indicating greater functional variation despite their high ANI values. These correlation differences– although numerically modest– support the earlier observation that key functional pathways are differentially present across strains, even within a highly conserved phylogenomic context.

## Discussion

The pangenome of 34 *Streptomyces albidoflavus* genomes revealed extensive genetic diversity, with only 3,867 genes forming the core genome and over 7,700 classified as an accessory (soft-core, shell, or cloud). This structure confirms the open nature of the *Streptomyces* pangenome, previously reported across the genus [23, 24], and reflects the ecological and functional versatility of *S. albidoflavus* strains. SM254 contributed uniquely to this diversity, carrying 185 strain-specific cloud genes and 1,346 shell genes. Such a high accessory gene content supports its distinct ecological niche and may underlie its reported antifungal activity against *Pseudogymnoascus destructans* [7].

The phylogenomic reconstruction based on 3,867 core genes placed SM254 within a tight clade of six closely related strains. Despite their high ANI (≥ 99%), SM254 displayed subtle divergence, especially in functional pathways. A separate group of four *S. albidoflavus* strains formed a highly divergent lineage, consistent with ANI values around 96%, suggesting possible species-level diversification or niche adaptation. This fine-scale resolution confirms that high nucleotide identity does not preclude meaningful ecological or metabolic differences [25], in line with recent insights into accessory genome-driven divergence in *Streptomyces* [26].

The ANI-based heatmaps further supported the genomic cohesion of the SM254 clade, while also highlighting that SM254 shared slightly lower identity values with most of its closest relatives than they did among themselves. These differences paralleled distinctions in pathway completeness, indicating that even near-clonal strains can vary in metabolism and ecological potential. ANI values above 99% typically denote functional equivalence [27, 28], yet our findings reaffirm that small-scale differences in accessory gene content can have disproportionate effects on phenotype [29, 30].

The functional annotation of SM254 revealed a metabolically robust strain. It encodes a complete TCA cycle, NADH-quinone oxidoreductase (98% completeness), F-type ATPase, Entner-Doudoroff pathway, and polyphosphate metabolism. These features reflect high respiratory capacity [31, 32], redox flexibility [33, 34], and energy conservation under phosphate-limited conditions [35]. The complete MEP/DOXP pathway supports the synthesis of isoprenoid precursors used in membrane [36], electron transport [37], and potentially secondary metabolites such as terpenes [38]. Although gluconeogenesis was absent, the presence of glycolysis and the TCA cycle compensates for carbon flow [39]. Polyphosphate metabolism, commonly associated with stress resilience and phosphate storage, may contribute to survival under oligotrophic cave conditions [31].

An in-depth comparison of functional profiles across the six closest strains revealed key differences in only a few pathways, most notably the complete presence of the ethanol fermentation pathway (“Mixed acid: Acetyl-CoA to Acetylaldehyde”) and the complete absence of asparagine biosynthesis in *S. albidoflavus* SM254. These two traits were exclusive to SM254 and defined its metabolic distinctiveness within the clade. Ethanol production, rare in *Streptomyces*, may offer an ecological advantage under oxygen-limited or biofilm-like conditions by enabling redox balance and generating antifungal byproducts such as ethanol and acetate, both known to inhibit fungal growth [40, 41]. A study by Jezewski et al., demonstrated that in fungi and bacteria, acetyl-CoA serves as a central building block for polyketide and fatty acid biosynthesis: enhancing its availability (via ethanol or acetaldehyde routes) can increase yields of acetyl-CoA-derived antifungal compounds [42]. Similar mechanisms have been reported in other bacteria using volatile organic compounds to suppress fungal pathogens [43, 44].

The absence of asparagine biosynthesis suggests auxotrophy, which is uncommon in *Streptomyces* but not unprecedented. Auxotrophs often emerge in environments where amino acids are abundantly available from external sources [45]. In the case of SM254, its sediment habitat may have contained decaying organic material or microbial biomass from which free amino acids such as asparagine could be scavenged. This ecological backdrop supports the hypothesis that SM254 has adapted to nutrient acquisition from its surroundings rather than investing in biosynthetic cost. The lack of asparagine synthesis may reroute glutamine/glutamate or other nitrogen precursors toward the biosynthesis of nitrogen-containing secondary metabolites [46]. For instance, a study by Chen et al., demonstrated that strains engineered for increased acetyl-CoA or lipid breakdown produced more polyketides and antibiotics due to reallocation of metabolic precursors [47].

Despite the overall functional similarity among the *S. albidoflavus* SM254 clade, the correlation network of pathway completeness values revealed subtle yet structured divergence. While most strains were nearly identical (correlation ≥ 0.99), SM254 displayed slightly lower similarity scores with others, primarily due to its unique metabolic profile. Such fine-scale divergence in functional potential, even in the absence of core genome variation, highlights the adaptive relevance of accessory pathways in shaping ecological interactions.

In summary, SM254 stands out from closely related *S. albidoflavus* strains due to its unique combination of metabolic versatility, secondary metabolite potential, and niche-specific adaptations. Its antifungal activity against *P. destructans* likely results from both classical antibiotic biosynthesis and metabolic traits such as fermentation and amino acid competition. The distinct genomic configuration of SM254 underscores how even subtle differences within highly similar genomes can translate into ecologically and functionally significant phenotypes.

## Conclusion

This study provides the first comprehensive comparative genomic analysis of *Streptomyces albidoflavus* SM254, a strain previously reported to antagonize the bat white-nose syndrome pathogen *Pseudogymnoascus destructans*. Despite high genomic similarity to other *S. albidoflavus* strains, SM254 exhibits unique metabolic features, including complete ethanol fermentation capacity and the absence of asparagine biosynthesis– traits not observed in its closest relatives. These findings, together with a metabolically versatile core genome, support the notion that SM254 is ecologically adapted to stress-prone, nutrient-variable environments like the Soudan Mine. While we cannot yet directly confirm the mechanisms underlying its antifungal activity, our results provide compelling genomic evidence for functional distinctiveness and potential for antifungal interaction. We hope this work will stimulate further biochemical and ecological investigations into SM254’s metabolic capabilities and its possible application as a natural biocontrol agent against *P. destructans*.

## Data Availability

The pipeline used for the bioinformatic data analysis has been deposited in GitHub: https://github.com/PopovIILab/AntiPd_SM254.
